# Sir Patrick Dunn's Hospital

**Published:** 1831

**Authors:** 


SIR PATRICK DUNN'S HOSPITAL.

MOTIONS AND SOUNDS OF THE HEART. Dr. NAGLE.

OUR readers are aware that we have not become converts to Dr. Corrigan's new theory of the heart's motions and sounds. We are not captious opponents; but ground our opposition on the evidence of our own senses—on pathology rather than on experiments. We have always been of opinion that PATHOLOGY would prove Dr. Corrigan to be wrong, and a case recently published by Dr. Nagle, in a contemporary journal, bears so much on this subject, that we shall place it before our readers.

*Case.* "Catherine Langan, aged 32, and married for 14 years, was affected with cough, dyspnœa, and palpitations of the heart, and, occasionally, with copious hæmoptysis. These symptoms commenced before her marriage, and recurred frequently, with more or less severity until the period of her coming under my observation. At that time, three months previous to her death, the pain in the region of the heart was extremely acute, the palpitations very violent, and the other symptoms much aggravated. Percussion elicited a dull sound over a large extent in the region of the heart, the actions of which could be heard all over the anterior part of the chest from the clavicles to the margin of the false ribs, especially on the left side. These were so rapid, tumultuous, and disorderly, as to preclude the possibility of any analysis until the remedies, usual for the alleviation of such symptoms, were employed. The heart's impulse was remarkably strong on the right and left of the sternum. The pulse, however, in the radial and other arteries was scarcely perceptible! No regular rhythm was, at any time, discoverable in the heart's movements. A number, six or eight perhaps, of comparatively weak and confused impulses followed each other in rapid succession, after which usually came about three—strong, rather regular, and admitting of an appreciable interval between them. The ear felt not then an alternation of soft and rigid impulses, as it did immediately before! The arterial pulse became, evidently, more regular and a little increased in strength during these few beats, but was by no means in proportion to the intensity of the impulse against the chest, with which it was, unquestionably, synchronous, and invariably exhibited the regularities and irregularities of the latter phenomenon. It was hard, wiry, and rapid. When the heart relieved itself, as it were, by these two or three comparatively slow and tranquil motions, then, and only then, a loud bellows-sound was audible between the ensiform cartilage and the margins of the eighth and ninth false ribs of the left side. This soufflet did not extend to the right side, but was traceable on the left to the extent of a few inches laterally from the sternum, and up to about the third rib, approaching which it exhibited an increased intensity. *Repeated observations left no doubt of its being perfectly synchronous with the impulse against the chest and the pulsations in the different arteries examined; and, what is important to observe, that it was substituted for the first of the two cardiac sounds. The contraction of the ventricles was attended with a loud dull sound on the right, with the bellows-sound on the left of the sternum.*

DIAGNOSIS.

From these and other particulars, less important and omitted for the sake of brevity, the following diagnosis was formed respecting the heart:—'Hypertrophy with dilatation of both ventricles, extending, in all probability, to the auricles. *Certainly*, an obstruction at the *aortic orifice*, occasioned either by disease of the valves or contraction of the aperture.' Having been asked by Dr. Clinton, who frequently visited with me this patient, and to whom I mentioned my diagnosis, if I considered the auriculo-ventricular orifices obstructed, I replied that 'there was no phenomenon to warrant even the suspicion of their being so; and that the result of a post-mortem examination would strongly confirm the reasonableness of my objections to the new doctrine respecting the heart's impulse against the chest:'—objections which he knew me to have repeatedly urged previous to the publication of Dr. Hope's papers. The patient, affected with general anasarca, dropsy, hydrothorax, &c., was admitted on the 4th of February last into Sir P. Dunn's Hospital, where she died in a day or two after. For the following description of the post-mortem appearances of the heart I am indebted to two very intelligent gentlemen, Dr. Nalty and Mr. Sharkey.

AUTOPSY.

Between the pericardium and heart there were no adhesions, nor any lymph effused; the pericardium contained about six ounces of fluid. The heart was enormously enlarged, the auricles and ventricles being exceedingly dilated and hypertrophied, but the hypertrophy in any did not appear proportioned to the dilatation. The hypertrophy and dilatation of both auricles were in proportion to those of the ventricles. The increased thickness seemed to extend to all the valves, which closed, perfectly, their respective apertures. *All* the orifices, except the aortic one, were not only quite free, but appeared to participate in the dilatation of the cavities. The *aortic aperture* was *very much* contracted, and its valves considerably more thickened than any of the others, which indeed were thought by some to be in a natural state, but adapted to their respective openings."

Dr. Nagle properly observes that, had he formed his diagnosis according to the principles of Dr. Corrigan's theory, he must decidedly have fallen into error. He would have attributed the bellows-sound to an obstruction at the auriculo-ventricular opening, as it occurred synchronously with the impulse against the chest. But the dissection proved that the obstruction existed in the aortic valves. The case is strongly subversive of Dr. Corrigan's theory.

